# Management of ADHD in Prisoners—Evidence Gaps and Reasons for Caution

**DOI:** 10.3389/fpsyt.2022.771525

**Published:** 2022-03-18

**Authors:** John Tully

**Affiliations:** School of Medicine, University of Nottingham, Nottingham, United Kingdom

**Keywords:** ADHD, prison psychiatry, impulsivity, aggression, comorbidities

## Introduction

Attention Deficit Hyperactivity Disorder (ADHD) is a neurodevelopmental disorder, estimated to affect 5–7% of children and adolescents worldwide [(1–3), though see (4, 5)]. ADHD was once thought to decline rapidly with age, with persistence into adulthood thought to be very uncommon (6). In the last two decades, thinking has shifted (7). While estimates for persistence into adulthood vary, more robust research studies suggest this is about 15% (8). Widely cited studies estimate the prevalence of adult ADHD in the general population to be 3–5% (9–11), with rates typically being higher for men, though concerns about overdiagnosis should be noted (5, 12–14). Increasing awareness and discussion about ADHD has led to development of several consensus and guidance documents internationally (15–21). In incarcerated populations, rates are thought to be disproportionately high (22), but underdiagnosed (23), leading to lobbying for prioritization of ADHD in prisons and calls for effective screening and treatment protocols (20–25).

Increased recognition and treatment of ADHD in adult prisoners has several potential benefits, beyond improvements in subjective well-being. Firstly, for many patients, receiving a diagnosis of ADHD may be validating (26). It could feasibly allow prisoners to come to terms with their offending histories and accept input from mental health services, which may in turn identify other mental health problems. Secondly, if treatment proves effective, resolution of core symptoms of the condition may encourage attendance at educational, occupational and therapeutic activities (25), which can have broader clinical benefits (27). Thirdly, if links between ADHD symptomatology and problematic behaviors within prisons, especially violence, are shown to exist, effective treatments would reduce the burden of these behaviors on prisons. Finally, as effective treatments for the most common cause of aggression and violence in prisoners—antisocial personality disorder (ASPD)—are very limited (28, 29), reattribution of some antisocial behavior to ADHD may have benefits to staff in mental health settings, reducing the therapeutic nihilism that is associated with ASPD (30, 31).

Efforts to improve outcomes for prisoners' mental health and reduce violence and offending are welcome. However, there are several reasons for caution toward the emergent focus on ADHD, and several gaps in the evidence base that need to be addressed. Below, I outline these in turn, offering suggestions for a measured and empirical approach to the condition within holistic formulation and management in prison psychiatry settings.

## Uncertainty About the Scale of the Problem in Prisons

Until recently, estimates of prevalence of ADHD in incarcerated populations varied so widely—from 4 to 72% (32)—as to resist meaningful interpretation. Hence, a 2015 meta-analysis of studies in prison populations (22) was beneficial. This found an overall prevalence of ADHD of 25.5% (26.2% in adult prisoners; no significant difference by sex) using diagnostic interviews.

This meta-analysis also revealed wide geographical variation in estimates, from 6.6% in Brazil (33) to 65.2% in Sweden (22). This could possibly be explained by differences in quality of assessments. Notably, studies included that used screening for diagnosis had a significantly higher estimated ADHD prevalence of 43.3% [though see (34)]. However, while a prevalence of about 25% was supported by a subsequent study in a Scottish prison using a structured interview (35), other high quality individual studies in male prisoners in Canada and France, using rigorous assessment protocols, report considerably lower prevalence rates of 17% (32) and 11% (36), respectively. These discrepancies may be explained in part by differential clinical profiles of prisoners between countries with large variation in sociodemographic profiles, though this explanation is perhaps less convincing for significant variation in rates between, for example, Scotland and France. It is also not clear to what extent a diagnosis of adult ADHD equates to clinically relevant symptomatology. A study in another Scottish prison (37) found a rate of 24% meeting criteria for ADHD in childhood, but only 23% of this subgroup (5.5% of the total sample) were fully symptomatic in adulthood at the time of the study (the study did not report how many were on treatment).

Overall, existing studies of prevalence ADHD in prisons continue to show considerable variation, even where more detailed assessments and stricter application of criteria are applied. Furthermore, receiving a diagnosis of ADHD does not necessarily equate to clinically relevant symptomatology. Together, this suggests ongoing caution is warranted in estimating the clinical impact of ADHD in any given prison population, and the need for ongoing rigorous assessments at national and local levels. As other authors have highlighted, inflated estimates of ADHD in prisons also risks stigmatizing individuals with ADHD in the general population as excessively prone to criminal behavior (38).

## Disproportionate use of Resources

A major shift in focus toward assessing and treating ADHD in prison risks a disproportionate approach to prison healthcare. Other mental disorders are very common in prisoners (39), with much higher rates of major depression (10–14%) and psychotic illness (4%) than in the general population (40, 41), and higher rates still in low and middle income countries (42). Substance misuse disorders are also very common (39, 43, 44), as high as 85% for male remand and 78% for male sentenced prisoners (43). Personality disorders are especially common, with estimates of 65% for any personality disorder and 47% for ASPD from a large-scale analysis (40). Borderline personality disorder [or emotionally unstable personality disorder (EUPD)] has been more commonly studied in female prisoners, with rates of about 25% (40), but may be similarly high in male prisoners (45). There are high rates of self-harm (46) and suicide (47, 48), which though linked to ADHD in general population (49), are strongly associated with personality disorders, depression, substance misuse, and psychosis (50–56), and of violence (57–59), which is especially associated with ASPD (56, 60–65). Despite this, prison mental health services remain chronically under-resourced (66), with substantial delays in transfer to hospital for treatment in most countries where data is reported (67–70), and limited access to and study of psychosocial and follow-up interventions that may be effective (27).

In this context, resources must be used judiciously, and proportionate to clinical need, in keeping with both standard procedures for allocation of community resources and the equivalence principle for prison healthcare (71). However, assessment of adult ADHD in accordance with good practice guidelines is heavily resource-intensive (16, 38). As ADHD is a neurodevelopmental disorder, therefore arising in childhood, confirmation of its presence in childhood and adolescence is essential to making a confident diagnosis of ADHD in adulthood. Yet confirmation of a diagnosis is often not available, requiring collation of collateral information from childhood. Additionally, in part due to concerns about drug-seeking and malingering of symptoms, good practice guidelines for adult ADHD (16, 17) appropriately call for a diagnostic assessment for adults, such as the DIVA (72), to be carried out, which takes a further 1.5 h. Assessments of adult ADHD therefore take significant additional time and resources, compared to assessment of common acute psychiatric presentations. This places further considerable pressures on mental health services in prisons. In the UK, guidance from the Royal College of Psychiatrists clearly states that ADHD is not an emergency (16), however in the clinical setting, services are coming under increasing pressure to rapidly assess and treat ADHD. For instance, a recent consensus statement highlights that “[commissioning groups] and clinicians are potentially at risk of being challenged if they ignore NICE Guidance and they should only do that if they have something better to offer” (20). Clinical teams must be apportioned reasonable timeframes to carry out assessments, and these should be aligned with available resources and the acute clinical need of other patients.

## Misattribution of Problem Behaviors

Excessive focus on ADHD may lead to misattribution of problematic behaviors. The large majority of individuals with ADHD do not offend (73). In those that do, a very high proportion have comorbid mental disorders. A meta-analysis demonstrated that in adult prisoners with ADHD, substance misuse disorders were comorbid in 74% of cases and personality disorders in 60% (74). A further selective review suggested the rate of comorbid mental disorder is as high as 96% (75). A broad interpretation of these figures would therefore suggest that it is these comorbid conditions, possibly alongside psychosocial factors, that would account for most of the offending in people with ADHD. This is supported by studies demonstrating no association between ADHD and criminal behavior when controlling for comorbid conditions such as conduct disorder and antisocial personality disorder [(76–79), although see (80, 81)], and a large epidemiological study of study in young people aged 16–24 demonstrating that the relationship between ADHD symptoms and offending among young people is largely explained indirectly by comorbid factors (77). Furthermore, the association between ADHD and criminality is reduced or eliminated with adjustment for lifetime substance use disorders (82, 83).

Despite this, a recurring theme in existing literature is the attribution of antisocial behavior in prisoners to ADHD. For instance, one editorial suggests that ADHD is “a major causal risk factor for the development of criminal behavior” (84). Another paper states that “the reasons for the particularly high rates of behavioral disturbance [in prisoners] with ADHD are likely to stem from several sources related to the core syndrome of ADHD, including impulsive responding, mood instability, emotional dysregulation and low frustration tolerance” (24). Yet emotional dysregulation is classified by DSM-5 only as an associated feature of ADHD, not a diagnostic specifier (15, 85). In contrast, it is a long-established core symptom of EUPD (borderline) personality disorder, which is present in up to 30% of prisoners (45). Despite theoretical explanations (86), whether the type of emotional dysregulation seen in ADHD is qualitatively different to that seen in EUPD or other disorders remains unclear (15). This raises the possibility that when emotional dysregulation is present in individuals with ADHD, it is mostly or always due to EUPD or other comorbid conditions, and not related to ADHD.

Likewise, aggression is not a diagnostic feature of ADHD. DSM-5 criteria for ADHD specify impulsive behaviors such as interrupting, blurting out answers, and difficulty waiting one's turn—not aggression or violence (85). In contrast, a low threshold for discharge of aggression, associated with impulsivity and low tolerance of frustration, is a core component of ASPD (87)—present in 47% of prisoners (40) and accounting for the large majority of violent crime in society (88–90). While plausible accounts of potential mechanistic links between ADHD and aggression and violence have been put forward (91–95), these remain theoretical. Notably, a meta-analysis investigating the neural underpinnings of cold and hot executive dysfunction in youth with disruptive behavior disorders (precursors of ASPD) found structural and functional deficits in relevant neural circuitry which were present irrespective of the presence of ADHD comorbidity (96). Hence, where antisocial behaviors, or traits, are present in prisoners with ADHD, they cannot be assumed to be *due* to ADHD.

In particular, to properly disentangle the relative contributions of ADHD and ASPD to violence and aggression in prisoners, there is a need for studies comparing those with ADHD and comorbid ASPD (ADHD+ASPD) to those with ADHD-only, and ideally, also those with ASPD-only, and healthy controls with neither condition. No such study has been carried out in adult prisoners. One study in a Scottish prison (37) showed that a small subsample of prisoners who were fully symptomatic or in partial remission for ADHD (10 ADHD-only, 17 ADHD+ASPD), were significantly more aggressive and functionally impaired than prisoners who were symptom free (103 no ADHD/ASPD, 68 with ASPD), after controlling for ASPD, using a sequential binomial logistic regression. However, no direct comparisons between ADHD-only, ADHD+ASPD, or ASPD-only were reported, and diagnosis of ASPD relied on MCMI profiles rather than a more rigorous assessment such as DSM criteria. In sum, the contribution of ADHD to aggression and violence over and above ASPD in prisoners has not been convincingly demonstrated to date, and should not be assumed to exist. As one of the arguments for treating ADHD in prisoners is reducing risk of aggression and violence (25), this must be factored into risk: benefit decisions about treatment.

## Unrealistic Expectations of Treatment

While expert guidelines state that the treatment of adults with ADHD should follow a multimodal approach, including psychoeducation and cognitive behavior therapy (15, 16), medication is now the first-line treatment for adults with ADHD (16, 17, 97). However, evidence for prescribing of stimulant and other medication in ADHD has been fraught with inconsistencies (98, 99) and beset by controversy (5, 100–102). Several meta-analyses have highlighted problems including lack of evidence for long-term effects, considerable incidence of adverse events, high risk of bias, and low to very low quality of evidence in studies of ADHD medications in both youths and adults (103–105). One meta-analysis found a poor benefit–risk balance for atomoxetine in adults with ADHD (106). Two other meta-analyses in adults (98, 99) showed no association between dose and efficacy of ADHD medications, raising questions about their mechanistic basis. The recommendation of medication as a first-line treatment for adult ADHD in the general population was made based on three randomized controlled trials, two of which were conducted by a group who came under investigation for undeclared conflict of interests (101). One Cochrane review—on immediate-release methylphenidate for adult ADHD (107)—was withdrawn in 2016 after substantial criticism of its methods and flawed conclusions (100). Taken together, this does not provide a clear-cut basis for prescribing medication in adult ADHD.

A more recent network meta-analysis (98) provided some support for use of stimulants, atomoxetine, and bupropion in adult ADHD. There were caveats however: evidence was found for short-term (12 weeks) effects only, there was a wide confidence interval (−0.99 to −0.58) for amphetamines, and medications were less efficacious and less well-tolerated in adults than in children and adolescents. Critically, trials in which participants had a comorbid disorder treated with non-ADHD medication were excluded (98). Given the high rates of mental disorder (40, 41) and use of psychotropic medication (108, 109) in prisoners, this undermines the generalisability of these findings to prisoners with adult ADHD. One randomized controlled trial provided evidence of improved global functioning following treatment with methylphenidate in prisoners with ADHD, though in a very small sample (*n* = 15 in treatment and placebo groups) and for a very short blinded observation period of 5 weeks (110). Another small RCT (111), in which outcomes in both placebo and treatment groups may have been confounded by simultaneous CBT, demonstrated reduced self-rated symptoms with methylphenidate compared to placebo, though no significant difference in clinician-rated improvement on CGI-I.

Taken together, existing trial data does not provide strong support for use of medications for ADHD in adult prisoners. Conduct of RCTs in prisons is especially challenging. An alternative to RCTs is pharmacoepidemiological studies, which allow evaluation of population-wide effects of medications. Evidence from one such large scale study based on Swedish registries of released prisoners (108) found a reduction in violent offending in those dispensed psychostimulants, though with a very wide confidence interval (within-individual hazard ratio 0.62, 95% CI 0.40–0.98). Another demonstrated significant reductions in the criminality rate in both men (32%) and women (41%), though sensitivity analyses (limited to men) showed that the rate reduction varied considerably (17 to 46%) depending on type of drug and type of outcome (112). Though the problem of confounding effects may be overcome with careful study design and appropriate sensitivity analyses, pharmacoepidemiological studies cannot cannot account for all possible confounders that select individuals to treatment and cannot prove causality. Validation with multiple samples and triangulation with other designs have been identified as a necessity (108). In particular, the absence of good evidence from adequately powered RCTs remains a concern, as has been repeatedly highlighted by NICE (18, 113). At least one such preregistered trial is now underway in the UK (113). This level of evidence is required to justify use of these medications as first-line interventions, the risks of which are discussed below.

## Risks of Prescribing

The risks of prescribing ADHD medication in prisoners are not trivial. Common or very common side-effects of methylphenidate and/or atomoxetine include aggression or hostility, anxiety/feeling jittery, abnormal behavior, depression and alterations in mood, and sleep disorders (114). This should be of particular concern in a population of patients with high baseline rates of all of these problems. Common or very common physical side-effects include arrhythmias, arthralgia, gastrointestinal disturbance, hypertension, and movement disorders (114). These risks are compounded by much poorer than average physical health in prisoners (115, 116). Furthermore, as many prisoners with adult ADHD will be treated with medications for comorbid conditions, potential drug-drug interactions must be considered (15). These include interactions between methylphenidate or atomoxetine and CYP 2D6 enzyme inhibitors such as fluoxetine, and increased risk of hypertension and other cardiovascular events through co-prescribing of agents such as duloxetine or venlafaxine (117).

Another important consideration is misuse of medication. Although modified-release formulations of stimulant medications are thought to reduce risk of misuse (118), all formulations carry a high risk for abuse and dependence if not used as prescribed (117). This is particularly important in prisoners, many of whom have extensive histories of substance misuse. Interaction of stimulants with other illicit drugs is also concerning. Illicit drugs remain a significant problem in prisons internationally (119–121), with synthetic cannabinoids (e.g., “Spice” and “Mamba”) a particularly troublesome issue in UK prisons (121, 122). Potential interactions of stimulants with other illicit drugs include a toxic sympathomimetic syndrome with prominent cardiac and neurological effects (123, 124). Evidence in human studies is mostly limited to a handful of small studies focused mainly on simultaneous use of alcohol (125), though one RCT showed that the hemodynamic and adverse effects of co-administration of methylphenidate and MDMA were significantly higher compared with MDMA or methylphenidate alone (126). It has been hypothesized that by reducing impulsivity and individuals' tendency to self-medicate, and addressing underlying mechanisms associated with addiction pathways, stimulant medication may help to protect against illicit substance use (23). However, such an effect in prison populations has not been demonstrated beyond a single small study, which was potentially confounded by simultaneous CBT (111). Notably, in meta-analysis in a general population sample, ADHD medications had no beneficial effect on drug abstinence (127). Diversion of prescribed stimulants—present in up to 80% of community samples (128)—is a further important consideration in prisons, where diversion and trading of many psychotropic medications remains a substantial problem (129, 130).

In sum, these findings provide reason for considerable caution in prescribing ADHD medications in prisoners. It has been suggested that not offering medication to prisoners with ADHD may be ethically questionable (131). However, the same is certainly true for providing any treatment to a vulnerable clinical population based on limited or substandard evidence, without due consideration of risks. These risks must be meaningfully weighed against potential benefits in all prescribing decisions.

## Conclusions

A focus on ADHD in prisoners has emerged in the last decade, with concerns that the condition is under-recognized and undertreated in prisons. Calls for a shift in emphasis toward ADHD are likely driven in part by exasperation with lack of effective treatments for other disorders, especially ASPD, which is exceptionally common in prisoners. There is much enthusiasm for developing new assessment and treatment pathways, alongside optimism that this will result in significant improvements in outcomes. To date, however, the evidence base to support all of this is very limited. Questions remain about the true prevalence of ADHD in prisoners, and the extent to which diagnosis equates with clinically relevant symptomatology. Particular concerns surround lack of evidence for mechanistic links between ADHD and antisocial behavior, misidentification of ADHD as a contributory cause of violence, inconsistent evidence for ADHD medication in adults generally (and almost no high quality evidence in prisoners), lack of cost-benefit analysis for interventions, and insufficient risk: benefit considerations in prescribing guidelines. To bridge these gaps, ongoing studies using robust assessment protocols are required to get a more accurate and granular understanding of rates on ADHD in specific prison populations. Randomized controls trials are required to support use of medication for ADHD as a first-line treatment. Importantly, given the prevalence of other mental disorders with direct links to self-harm, suicide, and violence, well-intentioned initiatives should not be allowed to create a disproportionate and misguided focus on ADHD as a primary problem in prison mental healthcare. Until a better standard of evidence exists, its status is more appropriately considered as under ongoing review.

## Author Contributions

The author confirms being the sole contributor of this work and has approved it for publication.

## Conflict of Interest

The author declares that the research was conducted in the absence of any commercial or financial relationships that could be construed as a potential conflict of interest.

## Publisher's Note

All claims expressed in this article are solely those of the authors and do not necessarily represent those of their affiliated organizations, or those of the publisher, the editors and the reviewers. Any product that may be evaluated in this article, or claim that may be made by its manufacturer, is not guaranteed or endorsed by the publisher.
